# LDL Cholesterol Variability Impacts the Prognosis of Patients with Chronic Ischemic Heart Disease: A Real-World Italian Experience

**DOI:** 10.3390/jcm12196231

**Published:** 2023-09-27

**Authors:** Pompilio Faggiano, Massimiliano Ruscica, Sara Bettari, Antonella Cherubini, Stefano Carugo, Alberto Corsini, Giulia Barbati, Andrea Di Lenarda

**Affiliations:** 1 Cardiovascular Department, Fondazione Poliambulanza, 25100 Brescia, Italy; cardiologia@pompiliofaggiano.it; 2 Department of Pharmacological and Biomolecular Sciences “Rodolfo Paoletti”, Università degli Studi di Milano, 20133 Milan, Italy; alberto.corsini@unimi.it; 3 Department of Cardio-Thoracic-Vascular Diseases, Foundation IRCCS Cà Granda Ospedale Maggiore Policlinico, 20154 Milan, Italy; stefano.carugo@unimi.it; 4 Spedali Civili of Brescia, 25123 Brescia, Italy; sara.bettari@live.it; 5 Cardiovascular Department, University and Hospital of Trieste, 34122 Trieste, Italy; antonella.cherubini@asugi.sanita.fvg.it (A.C.); dilenar@units.it (A.D.L.); 6 Department of Clinical Sciences and Community Health, University of Milan, 20122 Milan, Italy; 7 Biostatistics Unit, Department of Medical Sciences, University of Trieste, 34127 Trieste, Italy; gbarbati@units.it

**Keywords:** low-density lipoprotein cholesterol, atherosclerotic cardiovascular disease, statins, ezetimibe, revascularization, visit-to-visit variability

## Abstract

Epidemiologic, genetic, and clinical intervention studies have indisputably shown that low-density lipoprotein cholesterol (LDL-C) is causal in the development of atherosclerotic cardiovascular disease (ASCVD). However, LDL-C variability could be related to increased ASCVD risk in patients already treated with statins. The aim of the present retrospective real-life study was to assess the prognostic impact of LDL-C variability on all-cause mortality and cardiovascular hospitalizations in patients with stable cardiovascular artery disease. A total of 3398 patients were enrolled and followed up for a median of 56 months. Considering LDL-C < 70 mg/dL as the therapeutical target, during follow-up, the percentage of patients who achieved this goal raised from 20.7% to 31.9%. In total, 1988 events were recorded, of which 428 were all-cause deaths and 1560 were cardiovascular hospitalizations. At the last medical examination, each increase in LDL-C levels of 20 mg/dL corresponded to a 6% raise in the risk of any event (HR 1.06; 95%CI, 1.03 to 1.09). In conclusion, our real-world study supports the hypothesis that a continuous and progressive downward trend in LDL-C levels is needed to achieve and maintain a cardiovascular benefit, at least in secondary prevention.

## 1. Introduction

The past decade brought major advances in preventive cardiology that changed dramatically the landscape of prevention and treatment of atherosclerotic cardiovascular disease (ASCVD) [1]. Encompassing pathologies caused by atherosclerosis within the coronary, cerebral, and peripheral arteries and the aorta, ASCVD is a leading cause of death and disability worldwide [2]. As strongly supported by epidemiologic, interventional, and genetic studies, there is irrefutable evidence for the causal role of low-density lipoprotein (LDL-C) in ASCVD [3]. Thus, keeping LDL-C concentrations low to minimize the rate of progression of atherosclerotic plaques is a major strategy to reduce the risk of events [4]. Lowering LDL-C is beneficial in primary and secondary prevention with individuals at highest risk of atherothrombotic events (e.g., myocardial infarction, stroke, revascularization, or cardiovascular death), deriving greater absolute risk reductions [5].

Within this context, statins, a class of medications that reduce LDL-C levels, are a mainstay of primary and secondary ASCVD prevention [6,7,8]. An intensive statin dose, as compared with a moderate dose, incrementally lowers LDL-C and the rate of nonfatal cardiovascular events (e.g., in patients with acute coronary syndrome (ACS) or with stable coronary artery disease (CAD)) [9,10,11,12]. In last few years, there are more and more recommendations that for the selected highest risk patients, immediate combination therapy should be recommended, similarly to the combination therapy for patients with hypertension [13]. When added to statins, ezetimibe reduces further LDL-C levels by an additional 20 to 25% on average [14] and improves cardiovascular outcomes in ACS patients. According to the most recent 2023 European guidelines for the management of ACS, combination therapy with a high-dose statin plus ezetimibe may be considered during index hospitalization or it is recommended to intensify lipid-lowering therapy during the index ACS hospitalization for patients who were on lipid-lowering therapy before admission [15].

Although the relationship between adherence and treatment intensity is well known (i.e., the lowest cardiovascular risk is observed among adherent patients receiving high-intensity therapy) [16], a post hoc analysis of the TNT (Treating to New Targets) trial showed that in patients with stable CAD, the visit-to-visit LDL-C variability was an independent predictor of cardiovascular events [17]. Regardless of treatment effect and achieved LDL-C levels, the increase in LDL-C variability over time enhanced the risk of any coronary event by 16%, any cardiovascular event by 11%, death by 23%, myocardial infarction by 10% and stroke by 17%. Thus, not only the achievement of the therapeutic LDL-C goal but also a relatively constant trend of LDL-C levels during the follow-up of patients with coronary heart disease improves cardiovascular outcomes [17].

However, in clinical practice, only a small percentage of subjects achieves and maintains over time the therapeutic LDL-C target according to their own cardiovascular risk. Despite this awareness, a gap between clinical guidelines and clinical practice still stands. Alarming are the data of the DA VINCI [18] and SANTORINI [19] studies showing that, among European patients at high and very high risk for ASCVD, only a small percentage (roughly between 20 and 33%) reaches the LDL-C targets. This could be attributable to many factors (e.g., inappropriate doses of lipid lowering therapies, a switch toward lower doses of lipid lowering drugs, poor therapeutic compliance, and comorbidities) [20].

Thus, the aim of the present retrospective study was to assess how the visit-to-visit variability of LDL-C levels affected the achievement of the LDL-C therapeutic target (<70 mg/dL) in patients with stable CAD and how these changes were prognostic on all-cause mortality and cardiovascular hospitalizations.

## 2. Materials and Methods

### 2.1. Patient Population

This is an observational retrospective study based on data collected by the Outpatient Cardiology Department of University Hospital of Trieste and Brescia related to patients evaluated between November 2009 and October 2014. Follow-up was set at 31 December 2015. Patients with previous acute myocardial infarction, myocardial revascularization with either percutaneous coronary intervention or coronary artery by-pass grafting and patients with a diagnosis of coronary artery disease were included. The cardiovascular events needed for the enrolment had occurred at a median of 4 months (interquartile range: 2 to 15 months) before the first visit. Only patients who had at least 2 medical examinations with available information about LDL-C levels and lipid lowering therapies were extrapolated by the initial population and included in this dataset. The median number of medical examinations for patient was 2 (interquartile range: 2 to 4). Data relative to the first 6 follow-up visits were considered for each patient, for a final total amount of 3398 patients and 12,163 visits. During each visit, medical history, and the presence of traditional cardiovascular risk factors, such as type 2 diabetes mellitus, dyslipidaemia, arterial hypertension, age, smoking, and obesity, were considered. Pharmacological treatments were evaluated at each visit (angiotensin converting enzyme inhibitors (ACE-Is)), angiotensin II receptors blockers (ARBs), beta-blockers, statins, and antiplatelet drugs). Data relative to left ventricular ejection fraction and laboratory tests, such as serum creatinine, total cholesterol (TC), LDL-C and high-density lipoprotein (HDL)-C and triglycerides levels, were also collected. LDL-C was calculated by the Friedewald formula, which is as follows: LDL-C (mg/dL) = TC (mg/dL) − HDL-C (mg/dL) − triglycerides (mg/dL)/5. Echocardiography was performed with the patients in the left lateral decubitus using a 3.4 MHz transducer and left ventricular ejection fraction was calculated using the biplane Simpson method according to the international guidelines [21].

Over the course of the follow-up, the LDL-C average trend and the achievement of a goal of 70 mg/dL were recorded and also studied in association with other covariates (sex, age at each visit, a diagnosis of type 2 diabetes mellitus, a progressive number of visits, dyslipidaemia, current smoking). The cut-off of LDL-C < 70 mg/dL was chosen according to the therapeutic lipid lowering armamentarium that was available at that time (i.e., statins and statins plus ezetimibe) [14]. Moreover, the relationship between the LDL-C average trend and the lipid-lowering therapy were analyzed, evaluating if changes influenced the achievement of the target. The LDL-C variability was calculated for each subject at each visit (T) as the difference between the LDL-C value recorded at the visit (T0) and at the previous one at the visit (T-1), divided by the value of the visit (T-1). The LDL-C variability was calculated starting from the second visit for each patient included in the study, for a total amount of 8765 values.

The following outcomes were considered: all-cause mortality and cardiovascular hospitalizations both for acute coronary syndromes, and for revascularization with either percutaneous coronary intervention or coronary-artery bypass grafting. Data on fatal and nonfatal events have been obtained by direct or phone interview with patients and their families and hospital recording.

### 2.2. Statistical Analysis

Continuous variables are reported with mean ± standard deviation or median and IQR (interquartile range) as appropriate, depending on the distribution shape. Discrete variables are reported as absolute numbers and percentages. In order to analyze longitudinal trends of LDL-C values and the probability of reaching a target of <70 mg/dL, the following regression models were used: Linear Mixed Effects Models and Generalized Linear Mixed effect Models [22]. As random effects, we considered the centre (i.e., the hospital: Trieste or Brescia) assuming a random intercept for the centre, and the patient, in order to take into account the correlation between repeated measures from the same patient, with both random intercepts and slopes for the patient. To select significant factors for the multivariable models, a “nested models” procedure was adopted, by comparing the chi-square increment between nested models by adding one covariate at the time. For the prognosis, to evaluate the effect of longitudinal trends of LDL-C values and LDL-C visit-to-visit variability on the possible multiple events of hospitalizations and death, taking into account other time-dependent covariates measured across visits, a mixed effects Cox survival model using the gap time between visits/events as the time scale was estimated (using the R library “coxme”). Also in this case, a “nested models” procedure was applied to select parameters significantly associated with the outcome. Random intercepts were included in the model for “Centre” and “Patient”. The IBM-SPSS software version 19 (IBM, Armonk, NY, USA) and the R statistical software (The R Project for Statistical Computing, Vienna, Austria) were used for the analysis.

## 3. Results

### 3.1. Studied Population

A total of 3398 patients were deemed eligible to be enrolled into this retrospective analysis for a median follow-up of 56 months (interquartile range: 41–67 months) (Table 1).

The mean age was 69.9 years, with 32% being under 60 and 20% over 79. Total cholesterol was 171.9 mg/dL, LDL-C was 98.1 mg/dL, HDL-C was 47.9 mg/dL and triglycerides were 133.1 mg/dL. Most of the patients presented with arterial hypertension (81.6%) and dyslipidaemia (85.2%). The prevalence of other cardiovascular risk factors, such as smoking, obesity and type 2 diabetes mellitus was, respectively, 10.2%, 28.2% and 39.4%. Mean serum creatinine was 1.09 mg/dL and mean left ventricular ejection fraction was 54.9%. Before the enrolment, 68% of the patients had a myocardial infarction, 49% underwent percutaneous myocardial revascularization, 35% underwent surgical myocardial revascularization, and 30% had stable coronary artery disease. In the studied population, 76.6% were on ACE-inhibitors, 66.5% on beta-blockers and 91% on antiplatelet drugs. All patients were on statins for secondary prevention. Atorvastatin was the most prescribed (37.9%) statin, followed by simvastatin (34.2%), rosuvastatin (18.7%) and pravastatin (3%). The association simvastatin plus ezetimibe was given to 6.3% of patients. Only in 6% of the visits (363 patients), there was a switch to another type of statins.

### 3.2. LDL-C Average Trend and Achievement of the Therapeutic Target

**Figure 1:**
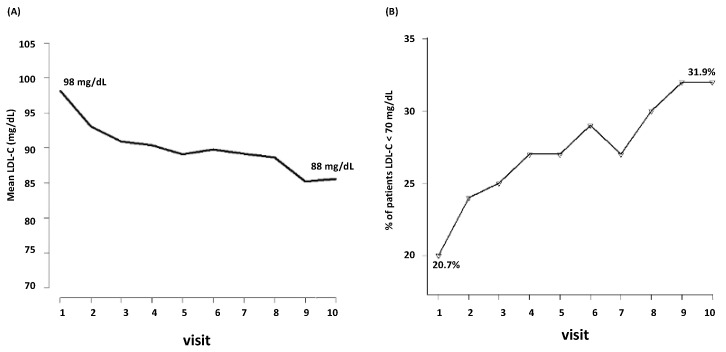
Visit-to visit changes in LDL-C (**A**) and achievement of LDL-C target (**B**). LDL-C, low-density lipoprotein cholesterol.

During the follow-up, LDL-C decreased from mean baseline levels of 98 mg/dL to 88 mg/dL (the tenth visit) (Figure 1A). Among the 3398 patients, only the 20.7% were at target (i.e., LDL-C < 70 mg/dL) at the first visit. During follow-up, the percentage of patients who achieved LDL-C < 70 mg/dL raised from the 20.7% to 31.9% (Figure 1B).

In the multivariable regression model (Table 2), male sex, older age, the diagnosis of type 2 diabetes mellitus, and a higher number of visits were associated with lower levels of LDL-C, whereas the diagnosis of dyslipidaemia and the tobacco use were correlated to higher LDL-C levels.

By using a logistic regression model, it was found that male sex, older age, and the diagnosis of type 2 diabetes mellitus were associated with a higher probability of achieving the therapeutic target (Table 3).

### 3.3. Prognosis

During the follow-up, 1988 events were recorded of which 428 were all-cause deaths and 1560 were cardiovascular hospitalizations. In the multivariable analysis, male sex, older age, arterial hypertension, tobacco use, and the diagnosis of type 2 diabetes mellitus were associated with higher rates of cardiovascular events. Furthermore, higher LDL-C levels at the visit preceding each outcome were significantly associated with a worse prognosis. At the last medical examination, each increase in LDL-C levels of 20 mg/dL corresponded to a 6% raise in the risk of any event (hazard ratio (HR) 1.06; 95% CI, 1.03 to 1.09; *p* < 0.001) (Table 4).

**Figure 2:**
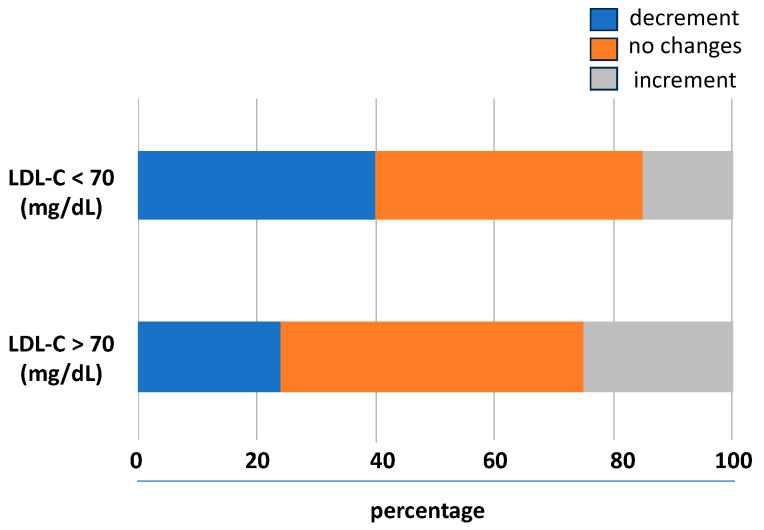
Visit-to-visit LDL-C variability trend during follow-up. LDL-C, low-density lipoprotein cholesterol.

The prognostic impact of the trend of LDL-C levels during the follow-up was studied analysing the association between the LDL-C variability and the outcomes. According to the baseline levels of LDL-C, its variability with a downward trend was associated with lower LDL-C levels and a higher rate to achieve values < 70 mg/dL. In the 31% of the follow-up visits, there was no significant relative LDL-C variability (i.e., two identical LDL-C values between consecutive visits); in 38%, there was a lowering of the LDL-C levels, and in the 31%, there was an upward trend. As shown in Figure 2, compared to patients not reaching the goal, in those achieving LDL-C < 70 mg/dL, a higher number of individuals had an LDL-C variability with a downward trend.

**Figure 3:**
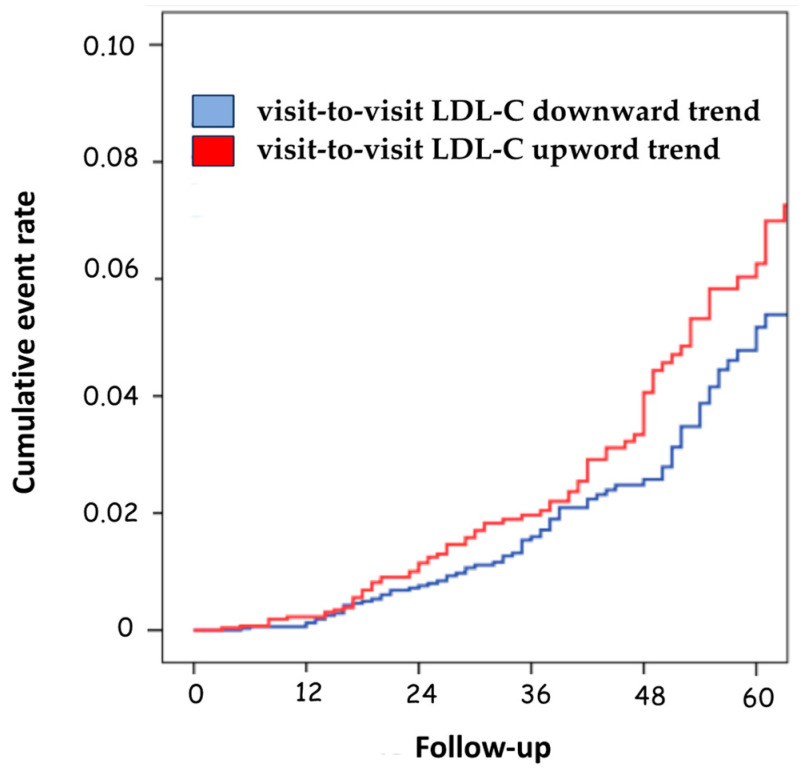
Cumulative event rate according to the visit-to-visit LDL-C variability trend. LDL-C, low-density lipoprotein cholesterol.

Only the LDL-C variability with a downward trend was associated with a significant decrease in the risk of negative prognostic events compared to patients with upward variability (HR 1.34; 95%CI, 1.10–1.90; *p* = 0.031) (Table 3 and Figure 3).

## 4. Discussion

The results of this retrospective study indicate that visit-to-visit LDL-C variability with a progressive and continuous downward trend was associated with a lower risk of cardiovascular events and all-cause mortality. Conversely, an upward visit-to-visit trend variability had a worse impact on the prognosis of patients with stable CAD. Indeed, it is well known that the benefit of lowering LDL-C is maintained up to very low levels of LDL-C. Based on this scientific evidence, current international guidelines recommend very demanding low LDL-C concentrations that generally cannot be obtained with statin monotherapy [23]. Every 38.7 mg/dL reduction in LDL-C is associated with a relative risk reduction of approximately 22% [24]. This association is maintained up to very low levels of LDL-C [25]. Of note, the risk reduction in major vascular events is independent of the starting LDL-C or the presence of diabetes or chronic kidney disease [26]. Beyond the paradigm “the lower the LDL-C the better”, it is important to consider the concept the earlier the reduction, the greater the cardiovascular benefit [27]. In fact, a threshold level has not yet been identified and a larger early LDL-C reduction and a more intensive statin therapy after myocardial infarction associate with a reduced hazard of all ASCVD outcomes and all-cause mortality in real-world setting [28]. An analysis of 135,688 patients on intensive lipid lowering therapies showed a linear correlation between absolute LDL-C reductions and the risk of major adverse cardiac events, with significant reductions in myocardial infarction (HR: 0.83, 95% CI 0.80–0.86) and stroke (HR: 0.81, 95% CI 0.75–0.87) [29].

Variability in LDL-C levels also may reflect behavioural or clinical factors that impair responsiveness to statins, the most obvious of which is inconsistent adherence to treatment. Fluctuating levels of LDL-C among patients treated with statins may parallel fluctuations in other biological processes influenced by therapy [30]. Reasons for not using life-saving lipid lowering therapies despite solid evidence of the benefits (e.g., statins) are complex and inadequately understood, regardless the fact that the safety of statins has been extensively demonstrated [31]. Besides that, insufficient intensification of lipid lowering therapy is in part due to physicians’ misperception on preventive LDL-C control [32]. Thus, our analysis suggested that long-term cardiovascular outcomes were increased in the group of patients with an upward variability. In the post hoc analysis of the TNT trial [17], Bangalore et al. demonstrated that each standard deviation increase of 1 in visit-to-visit variability in LDL-C was associated with a significant raise in any coronary event, cardiovascular event, death, myocardial infarction, and stroke in subjects with coronary artery disease. Overall, as assessed in a post hoc patient-level analysis of nine clinical trials involving 4976 patients with CAD who underwent serial coronary intravascular ultrasound, a greater visit-to-visit variability in atherogenic lipoprotein levels was significantly associated with coronary atheroma progression and clinical outcomes [33]. Interestingly, among the patients with non-obstructive CAD, a higher visit-to-visit LDL-C variability is associated with increasing all-cause mortality or composite endpoints during the long-term follow-up [34].

In line with this finding, a retrospective cohort study using the Clinical Practice Research Datalink showed that the lowest cardiovascular risk was observed among adherent patients receiving high-intensity therapy, while the highest one was observed among nonadherent patients receiving low-intensity therapy [16]. In a real-world setting, among 40,607 patients admitted for myocardial infarction and followed for a median of 3.78 years, larger and early LDL-C reduction and more intensive statin therapy were associated with a reduced hazard of all CV outcomes and all-cause mortality [28]. The findings of our analysis and the differences with the results derived from the post hoc analysis of the TNT trial could be explained by the different sample population of study. Data from our analysis belong to the experience of the real-world. Our population is very different from the one included in randomized controlled trials, for which the rigorous selection of the patients and the controlled and careful follow-up are the standard procedures. Instead, in clinical practice, the physicians often take care of elderly patients with many concomitant diseases, with poor medication adherence or polymedication or having trouble holding a regular follow-up. Relative to this, it is worth highlighting that in patients aged 75 years and older, lipid lowering is as effective in reducing cardiovascular events as it is in patients younger than 75 years [35]. The results of the meta-analysis of 28 trials with statins were similar, concluding that statin therapy produces significant reductions in major vascular events irrespective of age, but with less direct evidence of benefit among patients > 75 years old who do not already have evidence of occlusive vascular disease [36]. This is in line with the lower number of patients needed to be treated when patients > 65 years are considered [37,38,39]. Finally, among 82,958 simvastatin or atorvastatin initiators, statins were associated with a greater reduction in LDL-C levels in older persons than younger persons [40].

In the context of comorbidities increasing ASCVD risk, type 2 diabetes mellitus can not be overlooked. Indeed, estimates from epidemiological studies report that up to two- thirds of individuals with type 2 diabetes mellitus develop ASCVD in their lifetime, with many events attributable to ischemic heart disease [41]. This was consistent with our study reporting a 26% increase in the risk of hard outcomes in people diagnosed with type 2 diabetes mellitus. Thus, according to the 2023 European guidelines for the management of cardiovascular disease in patients with diabetes, statins remain the first-line therapy to reduce LDL-C levels in patients with diabetes and dyslipidaemia, due to their efficacy to prevent ASCVD events and to reduce cardiovascular mortality regardless of sex [42]. Finally, although hypertension is a well-known condition increasing cardiovascular risk, it is worth mentioning that in patients with a history of acute myocardial infarction, variabilities in LDL-C and blood pressure are powerful and independent predictors of cardiovascular events, including death [43].

Overall, the results of the present study should be interpreted within the context of potential limitations. First, the retrospective nature of our analysis and the occurring of the outcomes during the follow-up did not allow us to calculate only one mean value and standard deviation for all the medical examinations of each patient. However, we measured a variability value not for each patient, but for each single visit with respect to the previous one, clustered by patients. Thus, we have obtained values of visit-to visit variability and the chance of having available also the direction toward which the LDL-C levels tended to be modified compared to the previous measurement. The LDL-C variability was not reported as an absolute value, as calculated by the average absolute difference between two consecutive values but was calculated according to the upward or downward trend of LDL-C levels between two consecutive visits. After this consideration, our results could be better interpreted and compared to the post hoc analysis of data from the TNT trial. Second, since data were derived from a multicentric retrospective observational study, the measurement of lipid profiles was not performed in the same laboratory. Third, we were not able to evaluate differences among high- and low- or moderate-intensity statins or vs. the association statin plus ezetimibe. In particular, the choice of a specific statin (e.g., simvastatin in 34.2% of individuals) could be partly explained by reimbursement restrictions given by the Italian Medicine Agency (AIFA). Fourth, we were not able to report the percentage of patients suffering atrial fibrillation. However, it should be pointed out that atrial fibrillation is mainly related a higher absolute risk for heart failure [44]. Fifth, we did not perform any genotyping to confirm possible diagnoses of heterozygous familial hypercholesterolemia [45] and we did not evaluate any marker of endothelial function [46]. Sixth, the role of visit-to-visit variability of remnant cholesterol and HDL-C cannot be overlooked. Visit-to-visit remnant cholesterol variability values were associated with major adverse cardiac events in patients with type 2 diabetes independent of LDL-C [47] and high variability of HDL-C was associated with an increased risk of myocardial infarction, stroke, and mortality in the general population [48].

## 5. Conclusions

According to the current international guidelines, achieving very low levels of LDL-C in patients at high/very high cardiovascular risk is associated with fewer cardiovascular events and improvements in atherosclerotic plaques. Within this context, our real-world study supports the hypothesis that a continuous and progressive downward trend in LDL-C levels is needed to achieve and maintain a cardiovascular benefit, at least in secondary prevention. Physicians should be aware that variability in LDL-C levels may reflect behavioural or clinical factors that impair responsiveness to statins, the most obvious of which is inconsistent adherence to treatment [49]. Besides that, it should be highlighted that in patients aged 66 years or older who had a history of ASCVD and were on statins, adherence to treatment diminished progressively as baseline cardiovascular risk and future probability of death increased [50]. Thus, it is important to take advantage of the currently available lipid-lowering therapies, choosing the drug or combination of drugs that is most appropriate for each patient according to the cardiovascular risk and baseline LDL-C [23,51,52]. In our study, during the follow-up, the switching to other statins was performed in a small number of visits (6%) without affecting the achievement of the therapeutic target. This suggests that, despite the choice of switching to other types of statins, a lipid-lowering therapy with the same therapeutic power can be sufficient to maintain the therapeutic benefit.

## Figures and Tables

**Table 1:** Baseline characteristics of patients at the first visit. N = 3398.

	Value	Percentage
*Demographic characteristics*		
*Sex*		
Male	2442	71.9
Female	956	28.1
*Age (years)*		
	69.9 ± 9.4	
≤66	1107	32.6
67–73	978	28.8
74–78	678	20
≥79	632	18.6
*Blood biochemistry*		
Total cholesterol (mg/dL)	171.9 ± 43.3	
LDL-C (mg/dL)	98.1 ± 36.8	
LDL-C ≥ 70 mg/dL	2661	78.3
LDL-C < 70 mg/dL	703	20.7
HDL-C (mg/dL)	47.9 ± 13.3	
Triglycerides (mg/dL)	133.1 ± 72.9	
Serum creatinine (mg/dL)	1.09 ± 0.58	
*Echocardiographic parameters*		
Ejection fraction	54.9 ± 12.5	
*Comorbities*		
Hypertension	2773	81.6
Current smoker	345	10.2
Diabetes mellitus	1338	39.4
Dyslipidemia	2896	85.2
Obesity	958	28.2
*Statin therapy*	3398	100
Atorvastatin	1289	37.9
Pravastatin	101	3.0
Rosuvastatin	634	18.7
Simvastatin	1161	34.2
Simvastatin + ezetimibe	213	6.3
*Other medications*		
ACE-inhibitor	2604	76.6
Beta- blocker	2258	66.5
Antiplatelet drugs	3092	91.0

Data are expressed as means ± standard deviation.

**Table 2:** Multivariable regression model between LDL-C and covariates.

	Regression β Coefficient	SE	*p* Value
Sex (Males vs. Females)	−8.52	1.08	<0.001
Age (Increase of 5 years)	−1.4	0.05	<0.001
Diabetes mellitus	−8.20	0.84	<0.001
Progressive number of the visit (each)	−0.73	0.19	0.031
Dyslipidemia	13.20	1.27	<0.001
Current smoking	3.34	1.57	0.041

LDL-C, low-density lipoprotein cholesterol. SE, standard error.

**Table 3:** Logistic regression model between LDL-C target achievement and covariates.

	OR	95% CI	*p* Value
Sex (Male vs. Female)	2.36	1.80–3.11	<0.001
Age (Increase of 5 years)	1.11	1.05–1.18	0.002
Type 2 diabetes mellitus	3.10	2.45–3.92	<0.001
Dyslipidemia	0.22	0.16–0.31	<0.001

LDL-C, low-density lipoprotein cholesterol; OR, odds ratio; CI, confidence interval.

**Table 4:** Death from any cause and hospitalizations from cardiovascular causes.

	HR	95% CI	*p* Value
Age (Increase of 5 years)	1.15	1.12–1.18	<0.001
Sex (Male vs. Female)	1.16	1.04–1.27	0.01
Hypertension	1.14	1.00–1.31	0.05
Current smoker	1.17	1.00–1.37	0.05
Type 2 diabetes mellitus	1.26	1.15–1.39	<0.001
Increase in LDL-C (20 mg/dL) at the visit before the outcome	1.06	1.03–1.09	<0.001
LDL-C downward trend compared to upward trend	1.34	1.10–1.90	0.031

LDL-C, low-density lipoprotein cholesterol; HR, hazard ratio; CI, confidence interval.

## Data Availability

Data supporting reported results can obtained upon request to the corresponding author.

## References

[1] Wong N.D., Toth P.P., Amsterdam E.A., American Society for Preventive C. (2021). Most important advances in preventive cardiology during this past decade: Viewpoint from the American Society for Preventive Cardiology. Trends Cardiovasc. Med..

[2] Goyal A., Cho L. (2020). Preventive Cardiology and Risk Assessment: Beyond LDL. Curr. Atheroscler. Rep..

[3] Pirillo A., Casula M., Olmastroni E., Norata G.D., Catapano A.L. (2021). Global epidemiology of dyslipidaemias. Nat. Rev. Cardiol..

[4] Ference B.A., Graham I., Tokgozoglu L., Catapano A.L. (2018). Impact of Lipids on Cardiovascular Health: JACC Health Promotion Series. J. Am. Coll. Cardiol..

[5] Boren J., Chapman M.J., Krauss R.M., Packard C.J., Bentzon J.F., Binder C.J., Daemen M.J., Demer L.L., Hegele R.A., Nicholls S.J. (2020). Low-density lipoproteins cause atherosclerotic cardiovascular disease: Pathophysiological, genetic, and therapeutic insights: A consensus statement from the European Atherosclerosis Society Consensus Panel. Eur. Heart J..

[6] Grundy S.M., Stone N.J., Bailey A.L., Beam C., Birtcher K.K., Blumenthal R.S., Braun L.T., de Ferranti S., Faiella-Tommasino J., Forman D.E. (2019). 2018 AHA/ACC/AACVPR/AAPA/ABC/ACPM/ADA/AGS/APhA/ASPC/NLA/PCNA Guideline on the Management of Blood Cholesterol: A Report of the American College of Cardiology/American Heart Association Task Force on Clinical Practice Guidelines. Circulation.

[7] Mach F., Baigent C., Catapano A.L., Koskinas K.C., Casula M., Badimon L., Chapman M.J., De Backer G.G., Delgado V., Ference B.A. (2020). 2019 ESC/EAS Guidelines for the management of dyslipidaemias: Lipid modification to reduce cardiovascular risk. Eur. Heart J..

[8] Force U.S.P.S.T., Mangione C.M., Barry M.J., Nicholson W.K., Cabana M., Chelmow D., Coker T.R., Davis E.M., Donahue K.E., Jaen C.R. (2022). Statin Use for the Primary Prevention of Cardiovascular Disease in Adults: US Preventive Services Task Force Recommendation Statement. JAMA.

[9] Cannon C.P., Braunwald E., McCabe C.H., Rader D.J., Rouleau J.L., Belder R., Joyal S.V., Hill K.A., Pfeffer M.A., Skene A.M. (2004). Intensive versus moderate lipid lowering with statins after acute coronary syndromes. N. Engl. J. Med..

[10] de Lemos J.A., Blazing M.A., Wiviott S.D., Lewis E.F., Fox K.A., White H.D., Rouleau J.L., Pedersen T.R., Gardner L.H., Mukherjee R. (2004). Early intensive vs a delayed conservative simvastatin strategy in patients with acute coronary syndromes: Phase Z of the A to Z trial. JAMA.

[11] LaRosa J.C., Grundy S.M., Waters D.D., Shear C., Barter P., Fruchart J.C., Gotto A.M., Greten H., Kastelein J.J., Shepherd J. (2005). Intensive lipid lowering with atorvastatin in patients with stable coronary disease. N. Engl. J. Med..

[12] Pedersen T.R., Faergeman O., Kastelein J.J., Olsson A.G., Tikkanen M.J., Holme I., Larsen M.L., Bendiksen F.S., Lindahl C., Szarek M. (2005). High-dose atorvastatin vs usual-dose simvastatin for secondary prevention after myocardial infarction: The IDEAL study: A randomized controlled trial. JAMA.

[13] Ray K.K., Reeskamp L.F., Laufs U., Banach M., Mach F., Tokgozoglu L.S., Connolly D.L., Gerrits A.J., Stroes E.S.G., Masana L. (2022). Combination lipid-lowering therapy as first-line strategy in very high-risk patients. Eur. Heart J..

[14] Cannon C.P., Blazing M.A., Giugliano R.P., McCagg A., White J.A., Theroux P., Darius H., Lewis B.S., Ophuis T.O., Jukema J.W. (2015). Ezetimibe Added to Statin Therapy after Acute Coronary Syndromes. N. Engl. J. Med..

[15] Byrne R.A., Rossello X., Coughlan J.J., Barbato E., Berry C., Chieffo A., Claeys M.J., Dan G.A., Dweck M.R., Galbraith M. (2023). 2023 ESC Guidelines for the management of acute coronary syndromes. Eur. Heart J..

[16] Khunti K., Danese M.D., Kutikova L., Catterick D., Sorio-Vilela F., Gleeson M., Kondapally Seshasai S.R., Brownrigg J., Ray K.K. (2018). Association of a Combined Measure of Adherence and Treatment Intensity With Cardiovascular Outcomes in Patients With Atherosclerosis or Other Cardiovascular Risk Factors Treated With Statins and/or Ezetimibe. JAMA Netw. Open.

[17] Bangalore S., Breazna A., DeMicco D.A., Wun C.C., Messerli F.H., TNT Steering Committee and Investigators (2015). Visit-to-visit low-density lipoprotein cholesterol variability and risk of cardiovascular outcomes: Insights from the TNT trial. J. Am. Coll. Cardiol..

[18] Ray K.K., Molemans B., Schoonen W.M., Giovas P., Bray S., Kiru G., Murphy J., Banach M., De Servi S., Gaita D. (2021). EU-Wide Cross-Sectional Observational Study of Lipid-Modifying Therapy Use in Secondary and Primary Care: The DA VINCI study. Eur. J. Prev. Cardiol..

[19] Ray K.K., Haq I., Bilitou A., Manu M.C., Burden A., Aguiar C., Arca M., Connolly D.L., Eriksson M., Ferrieres J. (2023). Treatment gaps in the implementation of LDL cholesterol control among high- and very high-risk patients in Europe between 2020 and 2021: The multinational observational SANTORINI study. Lancet Reg. Health Eur..

[20] De Bacquer D., Astin F., Kotseva K., Pogosova N., De Smedt D., De Backer G., Ryden L., Wood D., Jennings C., Euroaspire I.V. (2022). Poor adherence to lifestyle recommendations in patients with coronary heart disease: Results from the EUROASPIRE surveys. Eur. J. Prev. Cardiol..

[21] Lang R.M., Bierig M., Devereux R.B., Flachskampf F.A., Foster E., Pellikka P.A., Picard M.H., Roman M.J., Seward J., Shanewise J.S. (2005). Recommendations for chamber quantification: A report from the American Society of Echocardiography’s Guidelines and Standards Committee and the Chamber Quantification Writing Group, developed in conjunction with the European Association of Echocardiography, a branch of the European Society of Cardiology. J. Am. Soc. Echocardiogr..

[22] Demidenko E. (2013). Mixed Models: Theory and Applications with R.

[23] Masana L., Plana N., Andreychuk N., Ibarretxe D. (2023). Lipid lowering combination therapy: From prevention to atherosclerosis plaque treatment. Pharmacol. Res..

[24] Cholesterol Treatment Trialists C., Baigent C., Blackwell L., Emberson J., Holland L.E., Reith C., Bhala N., Peto R., Barnes E.H., Keech A. (2010). Efficacy and safety of more intensive lowering of LDL cholesterol: A meta-analysis of data from 170,000 participants in 26 randomised trials. Lancet.

[25] Gaba P., O’Donoghue M.L., Park J.G., Wiviott S.D., Atar D., Kuder J.F., Im K., Murphy S.A., De Ferrari G.M., Gaciong Z.A. (2023). Association Between Achieved Low-Density Lipoprotein Cholesterol Levels and Long-Term Cardiovascular and Safety Outcomes: An Analysis of FOURIER-OLE. Circulation.

[26] Wang N., Fulcher J., Abeysuriya N., Park L., Kumar S., Di Tanna G.L., Wilcox I., Keech A., Rodgers A., Lal S. (2020). Intensive LDL cholesterol-lowering treatment beyond current recommendations for the prevention of major vascular events: A systematic review and meta-analysis of randomised trials including 327 037 participants. Lancet Diabetes Endocrinol..

[27] Ference B.A., Majeed F., Penumetcha R., Flack J.M., Brook R.D. (2015). Effect of naturally random allocation to lower low-density lipoprotein cholesterol on the risk of coronary heart disease mediated by polymorphisms in NPC1L1, HMGCR, or both: A 2 × 2 factorial Mendelian randomization study. J. Am. Coll. Cardiol..

[28] Schubert J., Lindahl B., Melhus H., Renlund H., Leosdottir M., Yari A., Ueda P., James S., Reading S.R., Dluzniewski P.J. (2021). Low-density lipoprotein cholesterol reduction and statin intensity in myocardial infarction patients and major adverse outcomes: A Swedish nationwide cohort study. Eur. Heart J..

[29] Cordero A. (2023). The efficacy of intensive lipid-lowering therapies on the reduction of LDLc and of major cardiovascular events. J. Clin. Lipidol..

[30] Baber U., Halperin J.L. (2015). Variability in low-density lipoprotein cholesterol and cardiovascular risk: Should consistency be a new target?. J. Am. Coll. Cardiol..

[31] Ruscica M., Ferri N., Banach M., Sirtori C.R., Corsini A. (2023). Side effects of statins: From pathophysiology and epidemiology to diagnostic and therapeutic implications. Cardiovasc. Res..

[32] Cosin-Sales J., Campuzano Ruiz R., Diaz Diaz J.L., Escobar Cervantes C., Fernandez Olmo M.R., Gomez-Doblas J.J., Mostaza J.M., Pedro-Botet J., Plana Gil N., Valdivielso P. (2023). Impact of physician’s perception about LDL cholesterol control in clinical practice when treating patients in Spain. Atherosclerosis.

[33] Clark D., Nicholls S.J., St John J., Elshazly M.B., Kapadia S.R., Tuzcu E.M., Nissen S.E., Puri R. (2018). Visit-to-visit cholesterol variability correlates with coronary atheroma progression and clinical outcomes. Eur. Heart J..

[34] Gu J., Yin Z.F., Pan J.A., Zhang J.F., Wang C.Q. (2019). Visit-to-visit variability in low-density lipoprotein cholesterol is associated with adverse events in non-obstructive coronary artery disease. Anatol. J. Cardiol..

[35] Gencer B., Marston N.A., Im K., Cannon C.P., Sever P., Keech A., Braunwald E., Giugliano R.P., Sabatine M.S. (2020). Efficacy and safety of lowering LDL cholesterol in older patients: A systematic review and meta-analysis of randomised controlled trials. Lancet.

[36] Cholesterol Treatment Trialists C. (2019). Efficacy and safety of statin therapy in older people: A meta-analysis of individual participant data from 28 randomised controlled trials. Lancet.

[37] Ruscica M., Macchi C., Pavanello C., Corsini A., Sahebkar A., Sirtori C.R. (2018). Appropriateness of statin prescription in the elderly. Eur. J. Intern. Med..

[38] Bach R.G., Cannon C.P., Giugliano R.P., White J.A., Lokhnygina Y., Bohula E.A., Califf R.M., Braunwald E., Blazing M.A. (2019). Effect of Simvastatin-Ezetimibe Compared With Simvastatin Monotherapy After Acute Coronary Syndrome Among Patients 75 Years or Older: A Secondary Analysis of a Randomized Clinical Trial. JAMA Cardiol..

[39] Lee S.H., Lee Y.J., Heo J.H., Hur S.H., Choi H.H., Kim K.J., Kim J.H., Park K.H., Lee J.H., Choi Y.J. (2023). Combination Moderate-Intensity Statin and Ezetimibe Therapy for Elderly Patients With Atherosclerosis. J. Am. Coll. Cardiol..

[40] Corn G., Melbye M., Hlatky M.A., Wohlfahrt J., Lund M. (2023). Association Between Age and Low-Density Lipoprotein Cholesterol Response to Statins: A Danish Nationwide Cohort Study. Ann. Intern. Med..

[41] Ruscica M., Macchi C., Giuliani A., Rizzuto A.S., Ramini D., Sbriscia M., Carugo S., Bonfigli A.R., Corsini A., Olivieri F. (2023). Circulating PCSK9 as a prognostic biomarker of cardiovascular events in individuals with type 2 diabetes: Evidence from a 16.8-year follow-up study. Cardiovasc. Diabetol..

[42] Marx N., Federici M., Schutt K., Muller-Wieland D., Ajjan R.A., Antunes M.J., Christodorescu R.M., Crawford C., Di Angelantonio E., Eliasson B. (2023). 2023 ESC Guidelines for the management of cardiovascular disease in patients with diabetes. Eur. Heart J..

[43] Bangalore S., Fayyad R., Messerli F.H., Laskey R., DeMicco D.A., Kastelein J.J., Waters D.D. (2017). Relation of Variability of Low-Density Lipoprotein Cholesterol and Blood Pressure to Events in Patients With Previous Myocardial Infarction from the IDEAL Trial. Am. J. Cardiol..

[44] Odutayo A., Wong C.X., Hsiao A.J., Hopewell S., Altman D.G., Emdin C.A. (2016). Atrial fibrillation and risks of cardiovascular disease, renal disease, and death: Systematic review and meta-analysis. BMJ.

[45] Sturm A.C., Knowles J.W., Gidding S.S., Ahmad Z.S., Ahmed C.D., Ballantyne C.M., Baum S.J., Bourbon M., Carrie A., Cuchel M. (2018). Clinical Genetic Testing for Familial Hypercholesterolemia: JACC Scientific Expert Panel. J. Am. Coll. Cardiol..

[46] Gutierrez E., Flammer A.J., Lerman L.O., Elizaga J., Lerman A., Fernandez-Aviles F. (2013). Endothelial dysfunction over the course of coronary artery disease. Eur. Heart J..

[47] Fu L., Tai S., Sun J., Zhang N., Zhou Y., Xing Z., Wang Y., Zhou S. (2022). Remnant Cholesterol and Its Visit-to-Visit Variability Predict Cardiovascular Outcomes in Patients With Type 2 Diabetes: Findings From the ACCORD Cohort. Diabetes Care.

[48] Han B.H., Han K., Yoon K.H., Kim M.K., Lee S.H. (2020). Impact of Mean and Variability of High-Density Lipoprotein-Cholesterol on the Risk of Myocardial Infarction, Stroke, and Mortality in the General Population. J. Am. Heart Assoc..

[49] Mann D.M., Glazer N.L., Winter M., Paasche-Orlow M.K., Muntner P., Shimbo D., Adams W.G., Kressin N.R., Zhang Y., Choi H. (2013). A pilot study identifying statin nonadherence with visit-to-visit variability of low-density lipoprotein cholesterol. Am. J. Cardiol..

[50] Ko D.T., Mamdani M., Alter D.A. (2004). Lipid-lowering therapy with statins in high-risk elderly patients: The treatment-risk paradox. JAMA.

[51] Ruscica M., Ferri N., Santos R.D., Sirtori C.R., Corsini A. (2021). Lipid Lowering Drugs: Present Status and Future Developments. Curr. Atheroscler. Rep..

[52] Ferri N., Ruscica M., Santos R.D., Corsini A. (2023). Fixed Combination for the Treatment of Dyslipidaemia. Curr. Atheroscler. Rep..

